# Pilot evaluation of the psychometric properties of a self-medication Risk Assessment Tool among elderly patients in a community setting

**DOI:** 10.1186/1756-0500-4-398

**Published:** 2011-10-11

**Authors:** Solomon J Lubinga, Ian Millar, Joseph B Babigumira

**Affiliations:** 1 Department of Pharmacy, Mbarara University of Science and Technology, Mbarara, Uganda; 2 Department of Pharmaceutical Sciences, Strathclyde Institute of Pharmacy and Biomedical Sciences, Glasgow, UK; 3 Global Medicines Program, Department of Global Health, University of Washington, Seattle, WA, USA

## Abstract

**Background:**

Although community pharmacists in the United Kingdom are expected to assess elderly patients' needs for additional support in managing their medicines, there is limited data on potentially useful assessment tools. We sought to evaluate a 13-item assessment instrument among community dwelling elderly patients, 65 years and above. The instrument is composed of a cognitive risk sub-scale of 6 items and a physical risk sub-scale of 7 items.

**Findings:**

The instrument was administered to elderly patients in a survey performed in a community to the west of Glasgow, Scotland. The survey recruited 37 participants, 31 from 4 community pharmacies and 6 patients whose medication management tasks were managed by the West Glasgow Community Health and Care Partnership (managed patients). Community pharmacists independently rated 29 of the 37 participants' comprehension of, and dexterity in handling their medicines. We assessed scale reliability, convergent validity and criterion validity. In sub-analyses, we assessed differences in scores between the managed patients and those recruited from the community pharmacies, and between multi-compartment compliance aid users and non-users. The instrument showed satisfactory internal consistency (Cronbach's alpha of 0.792 for 13-item scale). There was significant strong negative correlation between the cognitive risk sub-scores and community pharmacists' assessment of comprehension (ρ = -0.546, p = 0.0038); and physical risk sub-scores and community pharmacists' assessment of dexterity (ρ = -0.491, p = 0.0093). The Area Under the Receiver Operator Characteristic Curve (AUC ± SE; 95%CI) showed that the instrument had good discriminatory capacity (0.86 ± 0.07; 0.68, 0.96). The best cut-off (sensitivity, specificity) was ≥4 (65%, 100%). In the sub-analyses, managed patients had significantly higher cognitive risk sub-scores (6.5 versus 4.0, p = 0.0461) compared to non-managed patients. There was a significant difference in total risk score (4 versus 2, p = 0.0135) and cognitive risk sub-score (4 versus 1.5, p = 0.0029) between users and non-users of multi-compartment compliance aids.

**Conclusions:**

This instrument shows potential for use in identifying elderly patients who may have problems managing their own medicines in the community setting. However, more robust validity and reliability assessments are needed prior to introduction of the tool into routine practice.

## Background

Older people may have problems with self-management of their medicines and exhibit unintentional noncompliance, which may be caused by several risk factors such as a higher prevalence of chronic conditions, consumption of complex drug regimens, and age-associated physical, visual, and cognitive impairment [1]. And while studies suggest that social care support with medication management and the use of multi-compartment compliance aids improve adherence, clinical outcomes and quality of life [2,3], not all elderly people need such interventions; some are self-sufficient, and others are entirely reliant on these interventions [4]. For patients who need interventions, multi-compartment compliance aids may not be helpful in improving their drug management because dosage forms other than tablets and capsules may be unsuitable and patients with cognitive problems or blindness may ingest unit-dose blister packs [5,6]. Therefore, it is important to target interventions to those who need them.

The United Kingdom National Health Service Community Pharmacy Contractual Framework and the Disability Discrimination Act require community pharmacists to support self-care, provide a medicines assessment and compliance service and to support aspects of social care home services in medication management for the elderly and those with disabilities [7-10]. As such, community pharmacists should assess patients' needs for additional support in managing their medicines. Such an assessment would enable the pharmacist to determine exactly what support a patient would need: home delivery of prescriptions, reminder charts, large print labels, compartmentalised compliance aids, or social care support [8,11].

An evaluation of the Community Pharmacy Contractual Framework in England and Wales found that only 29% of pharmacies were providing medicines assessment and compliance service and 21% provided support to social care home services [7]. A recent study also found that very few patients received a systematic compliance aid assessment [12]. This may be because assessment tools and procedures tend to be complicated and the available tools have not undergone systematic validation in a community setting.

A brief task-based Risk Assessment Tool (RAT) was developed at the Department of Pharmaceutical Sciences, University of Strathclyde in Glasgow, Scotland. It was a 19-item instrument developed as a hospital pharmacy-based pre-discharge assessment for elderly patients and those recovering from stroke [[13,14]; Kusu-Orkar TG, unpublished data; Gallagher C, unpublished data]. In the studies of Meland E [unpublished data] and Yang F [unpublished data], in similar patient samples, it was refined to a 13-item tool, by dropping 6 items that had no informational value. The refined 13-item instrument was evaluated among rheumatoid arthritis patients [15]. We found only one study that evaluated the instrument in a community setting [Wen PC, unpublished data]. In this study, the instrument was evaluated among elderly patients (average age of 75 years) in community pharmacies. The 13-item tool however had no provision for assessing patients who were using eye/ear/nose drops. In addition, the study recruited participants who were "judged by the pharmacist to be able to handle the interview" and as such could have recruited low risk patients.

The present paper reports on further development of this instrument for use among elderly patients (65 years old and above) in community settings. We evaluated a modified version of the instrument with the aim of determining whether it is at least as good as an experienced community pharmacist, with knowledge of the patient, in assessing older patients' ability to manage their own medicines. Our primary objectives were to determine scale reliability and validate the instrument against community pharmacists' assessment of patients' ability to manage their medicines. Secondarily, we sought to compare RAT scores between patients receiving home care support with management of their medicines and other community dwelling elderly patients and between users and non-users of multi-compartment compliance aids.

## Methods

### Design and settings

We performed a 4-week survey in July and August 2008. We included participants who were at least 65 years old and taking at least 4 different medications daily and were either receiving social care support for administration of their medication, or using multi-compartment compliance aids, or both. These are important risk factors for non-compliance and problems with self-management of medications, and therefore represented an attractive group in which to test the RAT. Patients who did not consent to participate in the survey were excluded. We conveniently selected 4 community pharmacies in the west of Glasgow, Scotland from which participants were sampled. We also purposively selected participants whose medication tasks were managed (managed patients) by the '*Administration of Medicines*' scheme of the West Glasgow Community Health and Care Partnership. These were patients who had been assessed as unable to manage their own medicines and a social worker assumed responsibility for the medicines management tasks. Their medicines are dispensed in original packs with a Medication Administration Record chart that is completed daily by the carer after assisting the patient with his/her medicines.

### Procedures

We used a modified 13-item instrument with two sub-scales: 1) a cognitive risk sub-scale consisting of three comprehension questions, two motivation questions and one label-reading task and 2) a physical risk sub-scale consisting of six dexterity questions and one coordination question (Additional file 1). The Abbreviated Mental Test (AMT), a validated assessment of cognitive function among elderly patients [16] routinely used in clinical practice is embedded in our tool as the first item of the cognitive risk sub-scale. For each item, participants were assigned a score on an ordinal scale of 0, 1 and 2 in order of increasing difficulty. Total risk score was obtained by summing all questions in the instrument; cognitive risk sub-score, by summing questions 1 - 6, and physical risk sub-score by summing questions 7 - 13. A higher (worse) total score implied a higher risk of problems with self-management of medicines. In this study, item 13, the coordination question, was modified to include an assessment of the ability to administer eye/ear/nasal drops. In addition to manipulation of liquids on to a 5 ml spoon, participants who were using eye/ear/nasal drops regularly were asked to squeeze a drop of normal saline eye drops onto a cap. The assessment pack consisted of the following: 1) two 48 ml amber plastic bottles, one with a screw cap and the other with a child-proof cap; 2) a 100 ml glass bottle with a screw cap; 3) co-codamol 50/500 mg foil strip packs; 4) tegretol 20 mg blister packs; 5) 5 ml spoon; 6) normal saline eye drop and 7) a compliance aid (PlusPak^®^) filled with sweets. The labels were printed in Arial font, in 3 different sizes: 8, 10, and 12.

The primary author administered the instrument to consenting participants in the community pharmacies or their homes. Patients interviewed in pharmacies were a combination of appointments and opportunistic interviews with those using the pharmacy on the day the primary author was there. Community pharmacists set up interview appointments with suitable patients for the days the primary author was available in the pharmacy. Opportunistic interviews were initiated by having suitable patients identified by the pharmacist on the day the primary author was in the pharmacy. Patients interviewed in their homes included the managed patients and those identified by the community pharmacists for appointments who preferred to be interviewed in their homes. Suitable patients were defined as those who met our inclusion criteria, and had been under regular care of the community pharmacist for at least 6 months prior to the interview. These were participants for whom the pharmacist had good knowledge of their cognitive and physical ability to manage their medications. Managed patients who met our inclusion criteria were identified by pharmacists at the West Glasgow Community Health and Care Partnership and appointments were set up by telephone by the second author, a registered pharmacist in the United Kingdom.

Additional data on participant age, gender, living arrangements, medication management and multi-compartment compliance aid use were obtained from the participant and community pharmacy records.

Community pharmacists, who were blinded to the results of the instrument, were asked to independently rate each participant's comprehension of, and dexterity in handling their medicines on an ordinal scale of 1 to 4, in order of increasing ability. The four pharmacists (1 from each pharmacy) involved in rating our participants were experienced community pharmacists, each with at least 15 years of community pharmacy practice experience, and were the regular pharmacists in charge of the pharmacies from which our patients were recruited.

For criterion standards, participants were grouped into three "able" and "unable" categories defined as follows: 1) "able overall" (community pharmacists' score of 4 in both comprehension and dexterity assessments) and "unable overall" (score of less than 4 in either comprehension or dexterity assessments); 2) "able physically" (score of 4 in the dexterity assessment) and "unable physically" (score of less than 4 in the dexterity assessment); and 3) "able cognitively" (score of 4 in the comprehension assessment) and "unable cognitively" (score of less than 4 in the comprehension assessment). We opted to use the community pharmacists' assessment as our criterion standard because there was no alternative validated assessment tool in routine use in the participating community pharmacies.

### Statistical analysis

The data was entered into a database in Microsoft Excel 2007 and exported to STATA 10.0 (Stata Corporation, TX, USA) for analysis. Participant characteristics were summarised, continuous data as medians and Inter-quartile Ranges (IQR) and nominal data as percentages. Bivarite analyses were conducted to compare characteristics between managed participants and those whose medicine taking was not managed (non-managed) and between multi-compartment compliance aid users and non-users, focussing on the non-managed participants.

We used a Kruskal-Wallis test to determine whether there was a difference across ratings of participants' comprehension and dexterity by pharmacist. For scale reliability, Cronbach's alpha was computed for the total 13-item scale, physical risk and cognitive risk sub-scales. To investigate convergent validity, we used Spearman's correlation to determine the relationship between cognitive and physical risk sub-scores and community pharmacists' comprehension and dexterity scores respectively. Criterion validity was assessed by constructing Receiver Operator Characteristic (ROC) curves for the 13-item scale, and its component physical and cognitive risk sub-scales (against the respective criterion standards). We computed the Area Under the Curve ± Standard Error (AUC ± SE) and best cut-offs including sensitivity and specificity. The best cut-offs were defined as scores that produced the largest number of correctly classified participants.

Bivariate analyses were conducted to compare total risk score, cognitive and physical risk sub-scores between managed patients and non-managed participants and between multi-compartment compliance aid users and non-users. Bivariate analyses were performed using the two-sample Wilcoxon Rank Sum test for continuous data and the Pearson chi-squared test or Fisher exact test for categorical data.

### Ethics statement

All participants provided written informed consent. The Ethics Committee of the Department of Pharmaceutical Sciences, Strathclyde Institute of Pharmacy and Biomedical Sciences approved the study protocol. All procedures complied with the Declaration of Helsinki.

## Results

### Participant characteristics

Thirty-seven participants met the inclusion criteria and completed the interview. Of these, 8 were interviewed at home: the 6 managed patients and 2 others by preference. Of the 29 patients interviewed from the pharmacies, 6 (20.7%) were interviewed from the first two pharmacies each, 4 (13.8%) from the third pharmacy and 13 (44.8%) from the fourth. Overall, the median (IQR) age was 76 (72, 82) years and number of medicines taken was 8 (6, 10). About half (51.4%) of the participants were male. Twenty-two (59.4%) lived alone and of these 12 (54.5%) received routine social care support at their homes. The majority (70.3%) managed their medicines by themselves. Eighteen (48.6%) participants used multi-compartment compliance aids. Table 1 and Table 2 present participant characteristics factored for managed and non-managed patients and for multi-compartment compliance aid use (considering the 31 non-managed participants only) respectively. There were no significant differences in participant characteristics when these groups were compared.

**Table 1:** Profile of participants factored for medication management (N = 37)

Parameter	Managed (N = 6)	Non-managed (N = 31)	p-value
Age in years, median (IQR)	81 (77, 87)	76 (72, 80)	0.154
Number of regular medicines	8 (6, 9)	8 (6, 10)	0.984
Gender, n (%)			
Male	3 (15.8)	16 (84.2)	0.942
Female	3 (16.7)	15 (83.3)	
Living arrangement, n (%)			
Live alone	0 (0.0)	10 (100)	0.100
Live alone with help	4 (33.3)	8 (66.7)	
Do not live alone	2 (13.3)	13 (86.7)	
Use of multi-compartment compliance aid, n (%)			
Do not use	4 (15.8)	16 (84.2)	0.590
Use a dosett box	0 (0)	4 (100)	
Use the PlusPak^®^	3 (21.4)	11 (78.6)	

IQR - Interquartile Range

**Table 2:** Profile of non-managed participants (N = 31) factored for compliance aid use

Parameter	Users (N = 15)	Non-users (N = 16)	p-value
Age in years, median (IQR)	81 (77, 87)	76 (72, 80)	0.766
Number of regular medicines	8 (6, 9)	8 (6, 10)	0.339
Gender, n (%)			
Male	9 (15.8)	7 (84.2)	0.366
Female	6 (16.7)	9 (83.3)	
Living arrangement, n (%)			
Live alone	5 (50.0)	10 (50.0)	0.560
Live alone with help	5 (62.5)	8 (37.5)	
Do not live alone	5 (38.5)	13 (62.5)	
Daily management of medications at home, n (%)			
Self	11 (45.8)	13 (54.2)	0.311
Assistance from professional carer	2 (100)	0 (0.0)	
Assistance from relatives/friends	2 (40.0)	3 (60.0)	

IQR - Interquartile Range

### Overall RAT scores

Overall, total risk score, cognitive risk sub-score and physical risk sub-score ranged from 0-15, 0-9 and 0-7 respectively. Participants had a median (IQR) total risk score of 4.0 (2.0, 6.3), cognitive risk sub-score of 3.0 (1.0, 5.3) and physical risk sub-score of 0 (0, 2).

### Scale reliability and validity

Cronbach's alphas were 0.792, 0.679 and 0.813 for the 13-item, cognitive risk, and the physical risk sub-scales respectively. Dexterity items "Can open 48 ml amber plastic with screw cap" and "Can open 100 ml glass bottle with normal cap" were constant in our analysis sample and were therefore dropped from the analysis (Table 3). Further item analysis of the cognitive risk sub-scale showed that if motivation item 1, "Do you think your medicines are necessary for your health?" were removed, Cronbach's alpha rose to 0.712.

**Table 3:** Item-rest correlation and Cronbach's alpha if item deleted for the 13 item RAT (non-managed patients only, N = 31)

RAT Item	Item-rest correlation	Cronbach's alpha if item deleted
Abbreviated Mental Test	0.499	0.774
What medication do you take at the moment?	0.374	0.801
Have there been any changes in your medication recently?	0.517	0.774
Do you think your medicines are necessary for your health?	0.054	0.804
How confident are you about taking your medicines on your own?	0.229	0.797
Reading three sample labels of increasing font size (Arial: 8, 10, 12)	0.691	0.753
Can open 48 ml amber plastic with screw cap^a^	-	-
Can open 48 ml amber plastic with childproof cap	0.460	0.779
Can open 100 ml glass bottle with normal cap^a^	-	-
Can pop open blister packs	0.486	0.782
Can open foil strip of tablet	0.627	0.757
Manages to take out tabs from PlusPack^®^	0.531	0.784
Fine manipulation - 5 ml spoon and/or Dropper bottle	0.650	0.755

^a ^constant in study sample and dropped from the analysis

We obtained community pharmacists' assessments for the 29 (78.4%) participants who were interviewed in the pharmacies. There was no statistically significant difference in pharmacist ratings of participants' comprehension (χ^2 ^with ties = 1.453, p = 0.6932) and dexterity (χ^2 ^with ties = 1.724, p = 0.6316) by pharmacist. 15 (51.7%) participants were classified as "able cognitively", 16 (55.2%) as "able physically" and 9 (31%) as "able overall". There was a significant negative correlation between cognitive risk sub-score and the community pharmacists' assessment of comprehension (ρ = -0.546, p = 0.0038) and the physical risk sub-score and community pharmacists' assessment of dexterity (ρ = -0.491, p = 0.0093).

**Figure 1:**
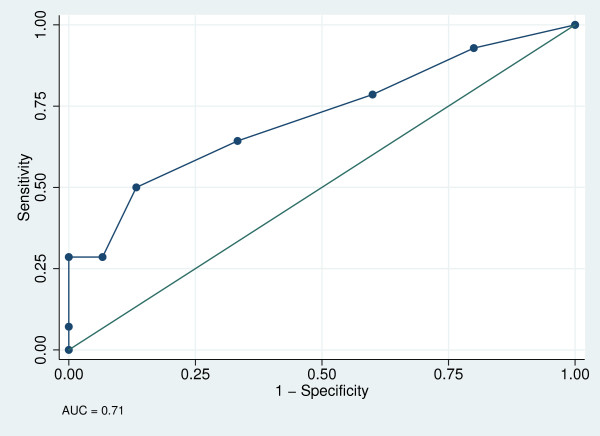
**Receiver operator curve for cognitive risk sub-scale**.

**Figure 2:**
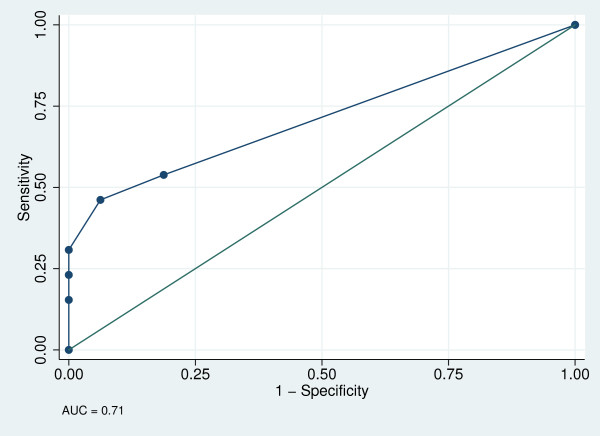
**Receiver operator curve for physical risk sub-scale**.

**Figure 3:**
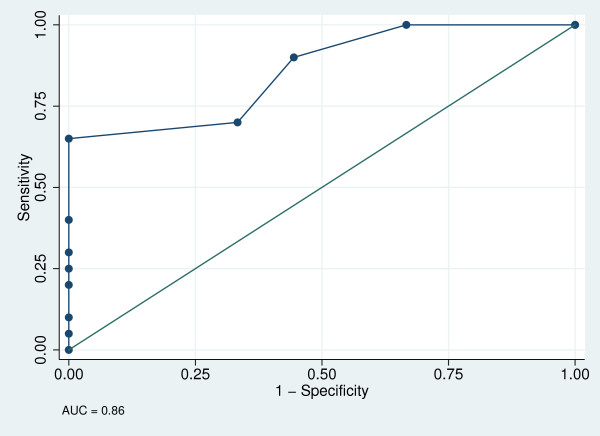
**Receiver operator curve for the overall Risk Assessment Tool**.

AUC ± SE (95% CI) were 0.71 ± 0.10 (0.53, 0.87) for the cognitive risk sub-scale (Figure 1), 0.71 ± 0.89 (0.53, 0.87) for the physical risk sub-scale (Figure 2) and 0.86 ± 0.07 (0.68, 0.96) for the total 13-item tool (Figure 3). The best cut-off points (sensitivity, specificity) were ≥4 (50.0%, 86.7%) for the cognitive risk sub-scale, ≥2 (46.2%, 93.8%) for the physical risk sub-scale, and ≥4 (65%, 100%) for the total 13-item tool, with 68.97%, 73.41% and 75.86% respectively, correctly classified.

### Comparative sub-analyses

Although managed participants had worse scores compared to the other community dwelling patients, only the cognitive risk sub-score difference was statistically significant (Table 4). The difference in total risk score approached statistical significance. The total risk score and cognitive risk sub-scores were significantly worse among multi-compartment compliance aid users compared to the non-users (Table 5).

**Table 4:** Comparison of scores between managed and non-managed participants

Parameter	RAT score, median (IQR)		p-value
		
	Managed (N = 6)	Non-managed (N = 31)	
Total risk score	6.5 (4, 12)	3 (2, 5)	0.0710
Cognitive risk sub-score	6.5 (4, 8)	3 (1, 4)	0.0461
Physical risk sub-score	1 (0, 3)	0 (0, 2)	0.4362

RAT - Risk Assessment Tool; IQR - Interquartile Range; managed patients - on managed level of care of the Administration of Medicines scheme of the West Glasgow Community Health and Care Partnership (WGCHCP)

**Table 5:** Comparison of RAT scores between multi-compartment compliance aid users and non-users (non-managed patients only, N = 31)

Parameter	RAT score, median (IQR)		p-value
		
	Users (N = 15)	Non-users (N = 16)	
Total risk score	4 (3.20, 6.80)	2 (1.00, 3.60)	0.0135
Cognitive risk sub-score	4 (3.00, 5.80)	1.5 (1.00, 2.60)	0.0029
Physical risk sub-score	0 (0.00, 1.80)	0 (0.00, 1.60)	0.7286

RAT - Risk Assessment Tool, IQR - Interquartile Range

## Discussion

### Overall RAT scores

Typical scores for the participants in our study were better than those in the prior studies that employed the instrument in elderly hospitalised patients (with or without stroke) and those with rheumatoid arthritis [[13-15]; Kusu-Orkar TG, unpublished data; Gallagher C, unpublished data; Meland E, unpublished data; Yang F, unpublished data]. These patients had worse total scores (>10) possibly because elderly hospitalised patients tend to be more debilitated than community dwelling patients. Additionally, stroke is commonly associated with both cognitive and physical impairment and rheumatoid arthritis is associated with significant dexterity problems. Though our scores were closer to those of Wen [unpublished data], who reported a median (IQR) total risk scores in patients recruited in community pharmacies of 2 (2, 3), they were slightly worse. This may be due to differences in study participant recruitment. The 6 (16%) managed participants, who had significantly worse total risk scores and cognitive risk sub-scores than the other participants, may have contributed to worse overall scores in our study. In addition, in the study by Wen [unpublished data], community pharmacists made a prior judgement on the ability of the participant to handle the interview as an inclusion criterion. As such, a degree of selection bias was unavoidable as the pharmacist could have selected participants who were 'able' physically and cognitively to participate in the interview, translating into better scores. In our study, a majority (29, 78.4%) of participants were recruited as they came into the pharmacies to collect their prescriptions, skipping the need for the pharmacists' judgement prior to the interview.

### Scale reliability and validity

Internal consistency was used as a measure of scale reliability. The Cronbach's alphas obtained for the 13-item scale and the physical risk sub-scale were above the indicative value of 0.70 for acceptable internal consistency [17]. If used alone, the cognitive risk sub-scale had a value lower that 0.70, indicating that it might not be reliable. However, internal consistency is improved by removal of motivation item 1, "Do you think your medicines are necessary for your health?" This item might be considered for removal if the cognitive risk sub-scale is to be used alone. Dexterity items "Can open 48 ml amber plastic with screw cap" and "Can open 100 ml glass bottle with normal cap" that were constant in our study sample should probably be considered for removal, as they carry no discriminatory value. This is consistent with the fact that all participants in our study successfully completed these tasks.

As with one previous study [Wen PC, unpublished data], a strong negative correlation between the instrument scores and the community pharmacists' assessment shows that there is agreement between the community pharmacists' assessments and the instrument. The ROC analysis (the AUC and 95% CI obtained) indicates that the full 13-item scale has good discriminatory capability. However, the wide 95% CI for the AUC for sub-scales with the lower bounds tending towards 0.5 imply that in some patients, the sub-scales used alone may not have good discriminatory capability. We found that a cut-off (sensitivity, specificity) total risk score of ≥4 (65%, 100%) would be the best value for screening purposes. The cut-offs obtained in our study were much lower than those in earlier studies among hospitalised elderly patients [13,14] and those with rheumatoid arthritis [15]. These conditions present unique cognitive and dexterity challenges to patients. In addition, while we used a community pharmacist's assessment as our criterion standard, these studies utilised a Global Clinical Assessment, which is a more commonly used assessment tool in the hospital setting. Though comparable, our 13-item cut-off is slightly higher than that of Wen [unpublished data] because our participants generally had worse scores.

### Comparative sub-analyses

Differences in scores between managed patients and other patients only resulted from the cognitive risk sub-scale. Managed participants had good physical risk scores, similar to other community dwelling elderly patients, implying that they are able to manage administration tasks just as well. Inability to self-manage medicines appears to have been the result of cognitive impairment (such as forgetfulness), which has been associated with unintentional non-adherence in the elderly [18,19]. Our results suggest that patients with poor scores on the cognitive domain of the instrument should be considered for social care support with medication management.

Only total risk score and cognitive risk sub-score were significantly worse for multi-compartment compliance aid users compared to non-users, suggesting that multi-compartment compliance aids should be considered in elderly patients with cognitive impairment, as a memory prompt, on condition that they are physically capable of self-administration. In this way, a patient who scores poorly on both physical and cognitive sub-scales would not be considered a candidate for multi-compartment compliance aid use and alternative means of medication support would be sought. However a patient who has poor scores on the cognitive sub-scale and good scores on the physical sub-scale would benefit from multi-compartment compliance aid use.

There are strengths inherent in the use of this instrument. It is a multidimensional instrument, covering several factors associated with the inability of patients to self-manage their medicines. The short time (15 to 20 minutes) it takes to conduct the assessment should be a welcome positive for practitioners as well as patients. Item 1, in the cognitive domain is based on a validated measure of cognitive function, the Abbreviated Mental Test [16]. The physical domain is strong as all items are based on completion of tasks related to actually taking medicines. In practice, it could help identify real physical limitations that patients may have in handling their medicines for example, failure to read standard size labels or opening child resistant caps, such that tailored interventions may be designed, for example, use of larger sized labels, replacing child resistant caps with ordinary screw caps. Generally, the instrument is easy to use and does not require pharmaceutical expertise to administer effectively. With a little training, social carers could use it to target appropriate support to those in need. They could use the instrument to bring both physical and cognitive limitations to medication management to the attention of pharmacists and other healthcare workers. It can also be used at the point of discharge from the hospital to help guide decisions about the most appropriate discharge destination as well as medication management support for individual patients.

### Study limitations

Our study was limited by a small sample size, which could have compromised our ability to make definitive statements on the psychometric properties of the instrument. In addition, the comparison of scores between managed and non-managed patients is limited by the fact that there were only 6 managed patients. Community pharmacists set up opportunist interviews and appointments. As such we did not gather the data how many they approached to make the 31 community participants; and we could only trace 6 managed participants through the WGCHCP records. And because we neither used a pre-validated instrument for the community pharmacists' assessments, nor conducted inter-rater reliability across pharmacists, further studies are needed to validate this tool against more robust instruments. We used a conservative definition of polypharmacotherapy (4 or more medicines). Our results showed that the median (IQR) number of medicines used by our patients was much higher, 8 (6-10), ranging from 4-21. Our study therefore might have been biased by lower risk patients in terms of the number of medicines taken. Although our study demonstrated an association between cognitive risk sub-scores and both multi-compartment compliance aid use and social care support with medicines management, there are no differences between patients in the physical risk domain of the tool. Therefore we are unable to determine, using the tool, exactly which patients would be candidates for either social care support or multi-compartment compliance aids. In spite of this, we found that typical scores in "managed" participants are worse than those who use multi-compartment compliance aids (6.5 versus 4.0). We can therefore discern that patients with much worse scores may be considered for social care support, as opposed to multi-compartment compliance aids.

There are limitations inherent in the tool itself. The questions concerning motivation ("do you think your medicines are necessary for your health?" and "how confident are you about taking your medicines on your own?") rely on the participant providing an honest answer. The question "what medications do you take at the moment?" assesses people's knowledge of their medicines. There are various dimensions to a patient's knowledge of their medicines, and the evidence linking knowledge of medicines to adherence and ability to self-manage medicines is at best conflicting [1].

## Conclusions

The instrument shows potential for use in identifying the limitations that older patients in the community may have with managing their medicines. With further modifications such as removal of items without discriminatory capacity, validation with a larger sample size and against more robust self-medication management assessment instruments, and determination of optimal cut-offs to distinguish between patients requiring home care medication management support or compliance aids, the tool could be considered for introduction into routine community practice.

## Competing interests

The authors declare that they have no competing interests.

## Authors' contributions

SJL participated in designing the study, collecting data, performing statistical analysis and drafting the manuscript. IM conceived the study, participated in designing the study, collecting data and revising the manuscript. JBB participated in performing statistical analysis and revising the manuscript. All authors read and approved the final manuscript.

## Supplementary Material

Additional file 1 **13-item self-medication Risk Assessment Tool**. Click here for file
