# Supplementary material for: Pilot evaluation of the psychometric properties of a self-medication Risk Assessment Tool among elderly patients in a community setting
Source: BMC Res Notes. 2011 Oct 11;4:398. doi: 10.1186/1756-0500-4-398 (PMC3199267; doi:10.1186/1756-0500-4-398)
Supplement: Additional file 1 — 13-item self-medication Risk Assessment Tool. [file 1756-0500-4-398-S1.DOC]

| **Abilities** | Tests | | **Risk Score** | | |
| --- | --- | --- | --- | --- | --- |
| Comprehension | 1 | Abbreviated Mental Test |  |  |  |
|  |  | - AMT (10/10) unimpaired | 0 |  |  |
|  |  | - AMT ≥ 7 partly impaired |  | 1 |  |
|  |  | - AMT < 7 impaired |  |  | 2 |
|  | 2 | What medication do you take at the moment? | 0 | 1 | 2 |
|  | 3 | Have there been any changes in your medication recently? | 0 | 1 | 2 |
| Motivation /insight | 4 | Do you think your medicines are necessary for your health? |  |  |  |
|  |  | - Yes, they are necessary | 0 |  |  |
|  |  | - Not sure |  | 1 |  |
|  |  | - They are not necessary |  |  | 2 |
|  | 5 | How confident are you about taking your medicines on your own? |  | | |
|  |  | - Confident | 0 |  |  |
|  |  | - Fairly confident |  | 1 |  |
|  |  | - Not confident at all |  |  | 2 |
| Reading labels | 6 | The patient will be tested with three sample labels of increasing font size |  | | |
|  |  | - Small font size (size 8) | 0 |  |  |
|  |  | - Medium font size (size 10) |  | 1 |  |
|  |  | - Large font size (size 12) |  |  | 2 |
| Dexterity |  | A kit of tablet and liquid packaging is used to test the patient’s skills |  | | |
|  | 7 | - Can open 48 ml amber plastic with screw cap | 0 | 1 | 2 |
|  | 8 | - Can open 48 ml amber plastic with childproof cap | 0 | 1 | 2 |
|  | 9 | - Can open 100 ml glass bottle with normal cap | 0 | 1 | 2 |
|  | 10 | - Can pop open blister packs | 0 | 1 | 2 |
|  | 11 | - Can open foil strip of tablet | 0 | 1 | 2 |
|  | 12 | - Manages to take out tabs from PlusPack® | 0 | 1 | 2 |
| Co-ordination | 13 | Fine manipulation - 5 ml spoon (and Dropper bottle for those who use eye or ear drops) | 0 | 1 | 2 |
| TOTAL | | |  | | |
